# Simultaneous Over-Expression of *PaSOD* and *RaAPX* in Transgenic *Arabidopsis thaliana* Confers Cold Stress Tolerance through Increase in Vascular Lignifications

**DOI:** 10.1371/journal.pone.0110302

**Published:** 2014-10-17

**Authors:** Amrina Shafi, Vivek Dogra, Tejpal Gill, Paramvir Singh Ahuja, Yelam Sreenivasulu

**Affiliations:** 1 Division of Biotechnology, CSIR-Institute of Himalayan Bioresource Technology, Palampur, Himachal Pradesh, India; 2 Academy of Scientific and Innovative Research (AcSIR), CSIR- Institute of Himalayan Bioresource Technology, Palampur, Himachal Pradesh, India; University of Delhi South Campus, India

## Abstract

Antioxidant enzymes play a significant role in eliminating toxic levels of reactive oxygen species (ROS), generated during stress from living cells. In the present study, two different antioxidant enzymes namely copper-zinc superoxide dismutase derived from *Potentilla astrisanguinea* (*PaSOD*) and ascorbate peroxidase (*RaAPX*) from *Rheum austral* both of which are high altitude cold niche area plants of Himalaya were cloned and simultaneously over-expressed in *Arabidopsis thaliana* to alleviate cold stress. It was found that the transgenic plants over-expressing both the genes were more tolerant to cold stress than either of the single gene expressing transgenic plants during growth and development. In both single (*PaSOD*, *RaAPX*) and double (*PaSOD* + *RaAPX*) transgenic plants higher levels of total antioxidant enzyme activities, chlorophyll content, total soluble sugars, proline content and lower levels of ROS, ion leakage were recorded when compared to the WT during cold stress (4°C), besides increase in yield. In the present study, Confocal and SEM analysis in conjunction with qPCR data on the expression pattern of lignin biosynthetic pathway genes revealed that the cold stress tolerance of the transgenic plants might be because of the peroxide induced up-regulation of lignin by antioxidant genes mediated triggering.

## Introduction

Abiotic stresses such as cold, drought, metal and salt stress are the major factors adversely affecting development and productivity in plants. Plants growing in different climatic niches have their own protective mechanisms to cope with the adverse conditions. Metabolic activities in various cellular compartments lead to the production of reactive oxygen species (ROS) such as superoxide anion (O_2_^−^), hydrogen peroxide (H_2_O_2_), hydroxyl radical (OH·), and singlet oxygen (O_2_) [1]. These ROS are highly toxic, and are taken care of by the antioxidant system in the living cells. During abiotic and biotic stress conditions, the production of ROS increases to lethal levels, however, there is an up regulation of both enzymatic and non-enzymatic antioxidants, which modify the ROS to non toxic forms. Oxidative stress response involving antioxidant enzymes includes superoxide dismutase (SOD), ascorbate peroxidase (APX), catalase (CAT), glutathione reductase (GR) and peroxidase (POD) [2]. SOD (EC1.15.1.1) is the first enzyme in the plant’s antioxidative defense mechanism that converts superoxide anion radicals (O_2_^.-^) to hydrogen peroxide (H_2_O_2_) and water, thereby imparting protection against the harmful effects of highly reactive superoxide radical (O_2_^.-^). APX is an integral component of the glutathione-ascorbate cycle [3] which detoxifies hydrogen peroxide in the presence of ascorbate to produce dehydroascorbate and water [4]. Genetic manipulation of antioxidant enzymes is one of the effective measures to impart stress tolerance in plants. Activities of superoxide dismutase and catalases were increased in plants subjected to cold stress [5]. It is commonly known that lignin evolved together with the plants adaptation to a terrestrial life to provide them with the structural support needed for an erect growth habit. Secondarily thickened cell walls were predominantly deposited by the lignin polymers, and making them rigid. In addition to developmentally programmed deposition of lignin, its biosynthesis can also be induced upon various biotic and abiotic stress conditions. The core enzymes of the phenylpropanoid pathway namely PAL, C4H and 4CL, converts the phenylalanine to *p*-Coumaroyl CoA. Thereafter, that the pathway flows to three directions, each of which is responsible for the production of individual components of monomers of lignin polymer i.e. *p*-hydroxyphenyl (H), guaiacyl (G) and syringyl (S) units. The generalized lignin biosynthetic pathway is shown in Fig. S1 and important enzymes which regulate the lignin pathway are also highlighted.

High altitude plants habitats are very specific because of their extreme niche areas that have naturally selected the best fit traits for adaptation in extreme environments. Their genes/proteins are being used as molecular tools for engineering crop and other plants for better stress tolerance and adaptability against the present scenarios of climate change. In *Potentilla atrosanguinea -* a Himalayan high altitude (4200 m msl) alpine plant, induction of a new copper-zinc superoxide dismutase (SOD) isozyme was recorded with the cold stress [6]. This gene encodes an isozyme of copper-zinc superoxide dismutase (EU532614.1) (*PaSOD*) and shared a homology of 80% (Expect = 5e-83) with the cytosolic *AtSOD* (NM_100757.3) [6]. Earlier studies confirmed that the protein encoded by this gene was functional from sub-zero temperature to >50°C and also the protein retained activity after autoclaving (heating at 121°C, at a pressure of 1.1 kg per square cm for 20 min) [7], [8]. Increased SOD activity has been positively correlated against different stress tolerance such as low/high temperature drought, salinity, high light intensity, and ozone [9], [10]. Over-expression of *Potentilla atrosanguinea* copper zinc superoxide dismutase (*PaSOD*) has improved copper and salt stress tolerance in *Arabidopsis thaliana* [11], [12]. Apparently, over-expression of the same SOD in potato, enhanced photosynthetic performance under drought stress [13]. In another study, over accumulation of lignin in vascular bundles was found to be the molecular mechanism which underlies the improved stress tolerance induced by the over-expression of *PaSOD* in *Arabidopsis thaliana* [14]. *Rheum australe* - another high altitude alpine plant, up regulation of ascorbate peroxidase was recorded against cold stress (unpublished data). This APX gene from Rheum (*RaAPX)*(DQ078123.2) was one of the cold tolerant genes, which shared a homology of 75% (Expect = 4e-162) with the peroxisomal *APX* gene of *Arabidopsis thaliana* (NM_119666.3). Hence it was thought that high altitude plants can be a good source of potential antioxident genes which can be successfully utilized for the reprogramming of stress tolerance in plants without disturbing native physiology, after functionally validated in the model plant i.e. *Arabidopsis*. In view of the above discussion, the present study illustrates the efficiency of over expressing two important antioxidant genes from high altitude plants namely superoxide dismutase from *Potentilla atrosanguinea* (*PaSOD*) and ascorbate peroxidase from *Rheum australe* (*RaAPX*) in *Arabidopsis thaliana*. It was established that simultaneous over expression of *PaSOD* and *RaAPX* was more effective in alleviating cold stress over independent over expression of either *PaSOD* or *RaAPX*.

## Materials and Methods

### Plant growth and cold stress treatment

*Arabidopsis* (ecotype *coloumbia*) plants were grown on soil mixture of vermiculite: peat moss: perlite (1∶1∶1) in the greenhouse under a 16 h light and 8 h dark cycle at 20±1°C. For stress treatment, 21d old seedlings of wild type, and hygromycin selected transgenic seedlings were transferred to cold room (4°C). Samples were collected 0 h, 6 h, 12 h, 24 h, 48 h (2d), 96 h (4d) and 192 h (8d) during cold treatment for the analyses of transcript levels, enzyme activity, proline and soluble sugars accumulation, electrolyte leakage, ROS, chlorophyll and biomass accumulation estimation etc,.

### Plasmid construction and plant transformation

Full length cDNAs of Copper-Zinc Superoxide Dismutase (*PaSOD*) and Ascorbate Peroxidase (now onwards *RaAPX*) from high altitude plants *Potentilla atrosanguinea* (which grows at day-time air temperatures of 3–10°C in Lahaul and Spiti districts of Himachal Pradesh: altitude 4517 m; 32° 24′ 20″N; 077° 38′ 40″ E) and *Rheum australe* (Rohtang Pass in Himachal Pradesh: altitude 4000 m; 32° 22′ 19″ N; 077° 14′ 46″ E), respectively, from Western Himalaya, were cloned in *Arabidopsis thaliana* as described earlier by Gill et al. [14]. Briefly, coding nucleotide sequences of these genes were amplified using the gene specific primers with incorporated *Nco*1 and *Bgl*II restriction sites at 3′ end. PCR products were cloned into a cloning vector pGEMT easy (Promega) and then sub-cloned into binary plant vector pCAMBIA1302 under the Cauliflower mosaic 35 S promoter. The prepared plasmid construct was mobilized into *Arabidopsis* plants via *Agrobacterium* mediated vacuum infiltration method [15]. Seeds were collected and screened in Murashige and Skoog [16] medium supplemented with 20 µg ml^−1^ hygromycin. Homozygous (T3 generation) transgenic *PaSOD* and *RaAPX* plants were crossed to obtain dual transgenic plants. Integration and expression of these transgenes in the dual transgenics was assessed by PCR using gene specific primers with genomic DNA as a template and semi-quantitative PCR from the total RNA isolated from leaf samples.

### Gene-specific semi-quantitative and real time PCR

Total RNA was isolated from 0 hrs and 2d cold treated transgenic and the wild type *Arabidopsis* plants using Total RNA extraction kit (Real Genomics). One microgram of total RNA was used for oligo (dT) primed first-strand cDNA synthesis in 20 µl reaction using of Superscript III Reverse transcriptase (Invitrogen). Transcripts of *PaSOD* and *RaAPX* were quantified with PCR using gene specific primers. Constitutively expressed *GAP-C* (Glyceraldehyde-3-phosphate dehydrogenase C subunit) was amplified simultaneously in 27 cycles to ensure equal amounts of cDNA used.

For real time expression analysis of lignin pathway genes during cold stress, primers were designed using Primer 3 software [17]. Details of the primers used in this study are given in Table S1. Gene expression was performed on a Stratagene Mx3000P system (Agilent Technologies, Germany) using 2× Brilliant III SYBR@ Green qPCR Master Mix (Agilent Technologies, Germany). All qPCRs were run in triplicates with a no-template control to check for contamination. PCR was conducted under the following conditions: 10 min at 95°C (enzyme activation), 40 cycles each of 30 s at 95°C, 30 s at 55°C and 72°C for 30 s and a final melting curve analysis was performed (55° to 95°C) to verify the specificity of amplicons. The raw threshold cycle (Ct) values were normalized against a housekeeping gene encoding Actin [18] to calculate both the difference in expression between control and respective treatment samples and tissue specific gene abundance using the Relative Expression Software Tool (REST; [19]). Expression values were transformed (log2) to generate expression profiles. This experiment was repeated three times for the reproducibility and for statistical significance calculation.

### SOD and APX enzyme activity assay

Total enzyme activity of SOD and APX was estimated at different time points during cold stress i.e. 0–192 h. Total SOD activity was estimated as described earlier [14] while APX activity was determined according to Nakano and Asada [20]. Briefly, leaf samples (100 mg) were homogenized in a pre-cooled mortar in homogenizing buffer containing 2 mM EDTA, 1 mM DTT, 1 mM PMSF, 0.5% (v/v) Triton-X100 and 10% (w/v) PVPP in 50 mM phosphate buffer pH 7.8. For APX activity homogenizing buffer contained ascorbate in addition and the buffer pH was 7.0. The homogenate was transferred to 1.5 ml Eppendorf and centrifuged at 13,000 rpm for 20 min at 4°C. The supernatant was used to estimate total SOD and APX activities. The total SOD activity was measured by adding 5 µl enzyme extract to a reaction mixture (200 µl) containing 1.5 µm Riboflavin, 50 µm NBT, 10 mM DL-Methionine and 0.025% (v/v) Triton-X100 in 50 mM phosphate buffer. One unit of enzyme activity was defined as the amount of enzyme required for 50% inhibition of NBT reduction at 25°C. Total protein content was estimated according to the dye binding method of Bradford [21] using BSA as standard. These experiments were repeated three times from three biological replicates.

### *In-situ* ROS staining

In situ ROS staining was done in accordance with Beyer and Fridovich [22], on the basis of the principle of NBT (nitroblue tetrazolium) reduction to blue formazan by O_2_^−.^ The intracellular concentration of ROS (O_2_^−.^) was directly proportional to the development of intensity of blue color in the leaves. Briefly, leaf tissue was detached from the wild type and transgenic plants and vacuum infiltrated with 10 mM sodium azide (NaN_3_) in 10 mM potassium phosphate buffer for 1 min. The infiltrated leaf tissue was incubated in 0.1% NBT (nitroblue tetrazolium) in 10 mM potassium phosphate buffer; pH 7.8 for 30 min. The stained leaf tissue was boiled in acetic acid:glycerol:ethanol (1∶1∶3) solution to remove other pigments and the stain content was visually documented under Carl-Zeiss Stereo DiscoveryV12 with Axiovision software. This experiment was repeated three times from three biological replicates.

### Estimation of electrolyte leakage

Cold-induced cell membrane injuries in leaf tissues were quantified by estimating the electrolyte leakage from the leaf samples. Electrolyte leakage was measured using an electrical conductivity meter as described by Lutts et al. [23]. Briefly, leaves were excised and washed with de-ionized water. One gram of fresh leaves were cut into small pieces (about 1–2 cm) and then immersed in 20 mL of deionized water, incubated at 25°C. After 24 h, electrical conductivity (EC1) of the bathing solution was recorded. These samples were then autoclaved at 120°C for 20 min to completely kill the tissues. Samples were then cooled to 25°C and the final electrical conductivity (EC2) of all released electrolytes was measured. The electrolyte leakage (EL) was expressed following the formula EL =  EC1/EC2×100. This experiment was repeated three times from three biological replicates.

### Estimation of total soluble sugars, proline and chlorophyll content

Total soluble sugar (TSS) was determined by anthrone method of Irigoyen et al. [24]. Free proline content was estimated using the acid ninhydrin method described by Bates et al. [25]. Fresh shoot and root samples (0.5 g) harvested at different time intervals were ground using mortar-pestle with 10 ml of 3% (w/v) sulfosalicylic acid aqueous solutions and the homogenate was filtered through Whatman no. 1 filter paper and taken for further analysis. Two ml of filtered extract, combined with 2 ml acid ninhydrin (1.25 g ninhydrin warmed, in a mixture of 30 ml glacial acetic acid and 20 ml of 6 M phosphoric acid, until dissolved). The reaction mixture was incubated in a boiling water bath (100°C) for 1 h and the reaction was then terminated in an ice bath. Then 4 ml of toluene was added to the reaction mixture and the organic phase (chromophore containing toluene) was warmed to room temperature and its optical density was measured at 520 nm using toluene as blank in a UV-visible spectrophotometer. The amount of proline was determined from a standard curve.

Chlorophyll content was estimated by the method of Arnon [26]. The chlorophyll from the fresh leaves was extracted in 80% acetone and the absorbance was read spectrophotometer (Synergy HT Multi-Mode Microplate Reader, Bio-Tek, USA) at 663 and 645 nm. These experiments were repeated three times from three biological replicates.

### Confocal and Scanning Electron Microscopy (SEM)

Confocal microscopy analysis was done as described earlier Gill et al. [14]. Stem of WT and transgenic lines were harvested and fixed in formalin, glacial acetic acid and 50% ethyl alcohol (FAA) (1∶1∶18) at room temperature. Samples were subsequently dehydrated in a tertiary butyl-alcohol series [27]; embedded in paraffin (melting point 58–60°C) and 8–10 mm sections were cut using a Finsee microtome. Sections were stained with 1% safranin in water and with 4% fast green in clove oil for 4 h and for 30 s, respectively. These were mounted in Canada balsam and examined using Confocal Laser Scanning Microscope (Zeiss LSM510 Meta Gmbh, Germany) equipped with a Zeiss Axiovert 100 M inverted microscope. Lignin auto fluorescence was collected by excitation/emission wave- lengths 488/505 nm.

For SEM analysis, stem cross-sections were fixed in two steps: first with a mixture of 2% paraformaldehyde and 2.5% glutaraldehyde in 0.1 mol/l cacodylate buffer, pH 7.4 for 1 h and then with 1% OsO_4_ in 0.1 mol/l cacodylate buffer, pH 7.4 for 30 m. After critical point drying, the samples were sputter-coated with gold, and the coated samples were viewed with a Hitachi S-3400N field emission SEM using an accelerating voltage of 30 kV.

### Statistical Analysis

All obtained data were analyzed statistically by Advance Linear/Non-linear models using General Linear Model (STATISTICA release 7, statsoft Wipro, Bangalore, Karnataka, India). Analysis of variance (ANOVA) followed by Duncan’s multiple range tests was used for the analysis of the results. The means of each treatment (as mentioned in respective experiments) and their interactions were compared and found statistically significant (*P*<0.05). The number of samples taken and their replicates are mentioned in respective experiments.

## Results

### Transgenic *Arabidopsis* over-expressing *PaSOD* in combination with *RaAPX* enhanced cold tolerance

**Figure 1:**
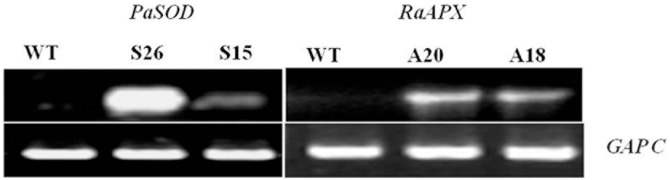
Confirmation of *PaSOD* and *RaAPX* gene expression in transgenic *Arabidopsis* plants at 20°C. The same plants were used for crossing to obtain the double transgenic plants. Expression of genes was (S26 and S15; A20 and A18) confirmed by estimating their transcripts from the leaf tissue by semi-quantitative RT-PCR. Constitutively expressed *GAP-C* was used as loading control.

**Figure 2:**
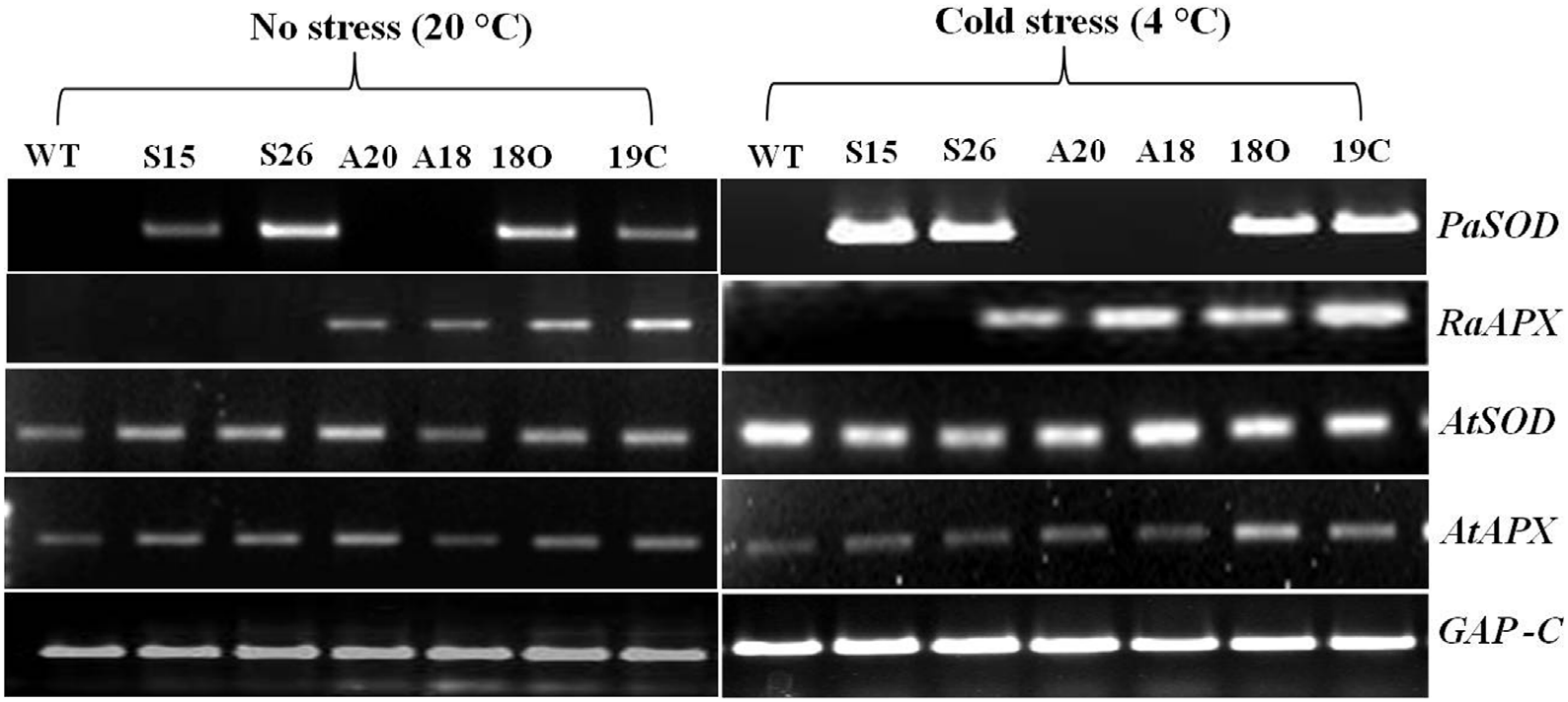
Assessment of native (*AtSOD*, *AtAPX*) and transgene (*PaSOD* and *RaAPX*) expression by estimating transcript levels under normal (20°C) and cold stress (4°C) conditions. Total RNA was isolated from the leaf samples of WT and transgenic plants, transcripts were amplified by using gene specific primers by semi-quantitative RT-PCR under normal and cold stress conditions. Constitutively expressed *GAP-C* was used as loading control.

*Arabidopsis* transgenic plants over expressing *PaSOD* under the control of CaMV35 S promoter were generated as reported earlier [12]. Similarly transgenic *Arabidopsis* plants over expressing ascorbate peroxidase gene from *Rheum* (*RaAPX*) under the control of CaMV35 S promoter were also developed. Presence of these transgenes was confirmed by PCR by using genomic DNA from these plants as template and their expression by the presence of its transcripts from leaf tissues by semi-quantitative RT-PCR (Fig. 1). On the basis of 3∶1 segregation and with high enzyme activities, homozygous transgenic lines S26 (for *PaSOD*) and A20 (for *RaAPX*) were selected for further analysis as well as for crossings for raising the transgenics with both these genes. Transgenic *Arabidopsis* plants having both *PaSOD* and *RaAPX* genes were obtained by crossings of these individual gene transgenic lines. Presence of transcripts for both genes in transgenic plants was confirmed by semi-quantitative RT-PCR (Fig. 2) and considered as transgenic line with both the genes. Two individual transgenic lines of each *PaSOD* (S15 and S26) and *RaAPX* (A18 and A20) were analysed and data was presented. Out of a number of transgenic lines having both the genes, the line 18O and 19C was selected for further analysis and data was presented.

**Figure 3:**
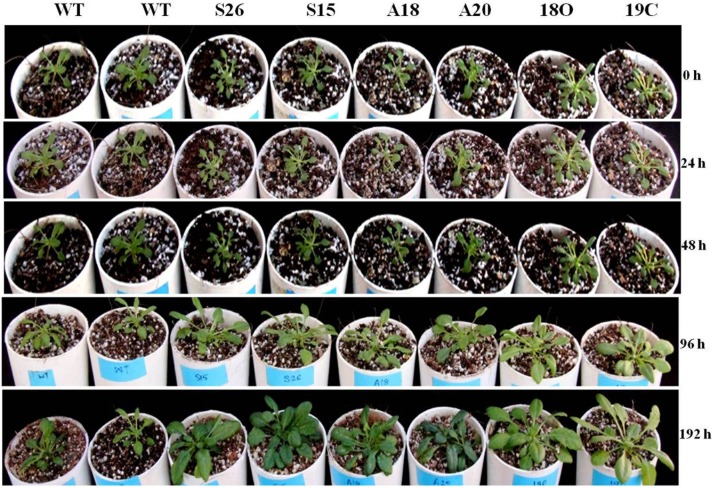
Transgenic *Arabidopsis* plants showing tolerance to cold stress. Five weeks old seedlings were transferred to cold (4°C) and allowed to grow for 192 h and different stress tolerance parameters were estimated from the samples of these plants.

Thus generated single and double transgenics *Arabidopsis* plants were found tolerant when exposed to cold (4°C) stress (Fig. 3). The morphological and vegetative growth of transgenic plants was more or less similar to the wild plants under normal growth conditions i.e. 20±1°C and 16 h/8 h light regime (Fig. S2) whereas, under cold stress the transgenic plants showed better growth (Fig. 3). Effects of cold stress were clearly observed in the growth of wild type plants, where leaves started yellowing which shows the symptoms of chlorosis with stunted growth but the transgenic plants showed better growth. *PaSOD* single transgenic plants (S26 and S15) and double transgenic plants (18O and 19C) were better than the *RaAPX* transgenic plants (A20 and A18). The same was confirmed by the accumulation of transcripts levels also (Fig. 2). *PaSOD* (S26, S15) and *RaAPX* (A20, A18) transgenic lines exhibited expression of *PaSOD* and *RaAPX*, respectively, whereas 18O and 19C exhibited expression of both genes under normal conditions. A low level of transcripts was observed for plants without stress at 20°C. Further, significant over accumulation of transcripts of transgenes were observed in single as well as in double transgenic plants under cold stress (Fig. 2).

### *PaSOD* and *RaAPX* induced cold stress tolerance alters *Arabidopsis* root growth and biomass

**Figure 4:**
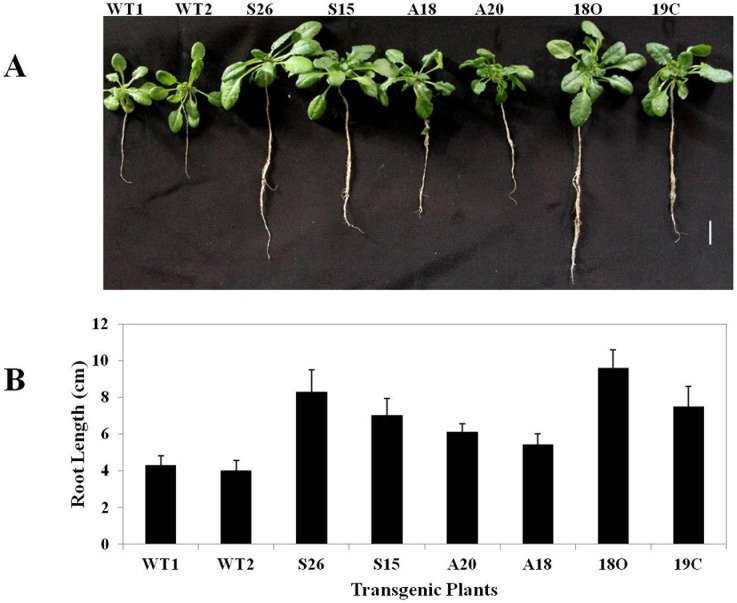
Root growth of WT and transgenic plants under cold stress. A). Photograph showing root growth of transgenic plants after cold stress (192h). B). Graphical representation of root length of transgenic plants after cold stress (192 h). Values are the representation of Mean±SE of three biological replicates. Transgenic plants developed better root system and thus exhibited better stress tolerance.

Root growth and development pattern was also studied during cold stress. After 192 h of cold stress in the single PaSOD transgenics (S26, S15) and double transgenic lines (18O, 19C) showed increased root growth biomass (Fig. 4). But in A20 and A18 the APX transgenic Arabidopsis plants showed decreased root growth when compared to WT. In order to determine if this difference existed in young seedlings at the in vitro stage, root growth on normal medium without any stress was also measured. A marginal increase in root length was observed in between WT and transgenic plants under normal growth conditions (Fig. S3). Initially, WT and transgenic plants exhibited similar root growth under cold stress, but WT root growth rate was lower than that of transgenic, which had longer roots after a prolonged culture period (10 d). Transgenic plants efficiently tolerated the stress and exhibited better root growth and biomass production in cold stress conditions (Table 1). Transgenic *Arabidopsis* plants over expressing *RaAPX* perform more or less similar in certain aspects of vegetative growth like rosette diameter, number of leaves; reproductive growth in terms of number of pods, length and number of seeds per silique, with the transgenic over expressing both *PaSOD* and *RaAPX* genes under cold stress conditions (Table 1). But overall, the transgenic plants over expressing both the genes performed better under cold stress.

**Table 1:** Growth and biomass production under normal (20°C) and cold stress (4°C) conditions in WT and transgenic plants. ±mean SD values.

Plant type	No of leaves		Rosettediameter (cm)		Plant height(cm)		No of pods		Pod length(cm)		Total seeds	
	20°C	4°C	20°C	4°C	20°C	4°C	20°C	4°C	20°C	4°C	20°C	4°C
**WT**	**18.6±1.2^c^**	**19.4±1.2^c^**	**3.9±0.4^d^**	**3.3±0.3^d^**	**26.5±1.07^ef^**	**20.8±0.4 ^g^**	**43.6±1.1^de^**	**31±1.0 ^g^**	**1.14±0.1^fghi^**	**1.08±0.1^fghi^**	**884±14.7^d^**	**620±20.0 ^g^**
***PaSOD*** ** (S15)**	**25.6±1.6^ab^**	**25.4±0.6^ab^**	**5.5±0.2^bc^**	**6.6±0.2^a^**	**27.3±0.94^cde^**	**31.8±0.9^ab^**	**39.2±0.3f**	**56.4±0.5^c^**	**1.5±0.1^bcd^**	**1.62±0.1^abc^**	**784±7.4^f^**	**1128±10.2^c^**
***PaSOD*** ** (S26)**	**24.2±2.0^b^**	**24±1.1^b^**	**5.3±0.4^c^**	**6.72±0.1^a^**	**26.5±1.28^ef^**	**31.4±1.1^ab^**	**37.4±0.9^f^**	**58.2±1.0^c^**	**1.38±0.1^cde^**	**1.68±0.1^abc^**	**748±18.5^f^**	**1164±21.3^c^**
***RaAPX*** ** (A20)**	**17.4±0.6^c^**	**27.8±.7^ab^**	**5.4±0.2^c^**	**6.9±0.2^a^**	**23.1±1.12^fg^**	**30.2±1.3^bc^**	**43.2±1.0^de^**	**68.4±0.9^ab^**	**1.22±0.1^fgh^**	**1.82±0.1^ab^**	**864±20.4^de^**	**1368±8.5^ab^**
***RaAPX*** ** (A18)**	**18.2±0.3^c^**	**29±1.0^a^**	**5.5±0.2^bc^**	**6.68±0.2^a^**	**23.7±0.99^efg^**	**30±1.4^bcd^**	**41.6±0.6^e^**	**67.2±0.3^b^**	**1.3±0.0^efg^**	**1.94±0.1^a^**	**832±12^e^**	**1344±7.4^b^**
***PaSOD*** ** + ** ***RaAPX*** ** (18O)**	**20±1.3^c^**	**28.4±0.5^a^**	**5±0.4^c^**	**6.9±0.5^a^**	**25.8±0.73^ef^**	**34±1.7^a^**	**42.8±0.6^de^**	**70.6±1.0^a^**	**0.94±0.0^hi^**	**1.78±0.1^ab^**	**856±13.2^de^**	**1412±21.5^a^**
***PaSOD*** ** + ** ***RaAPX*** ** (19C)**	**20.4±1.2^c^**	**27.2±0.9^ab^**	**5.2±0.3^c^**	**6.5±0.3^ab^**	**26.6±0.68^ef^**	**33.2±1.3^ab^**	**43.6±0.6^de^**	**69.4±0.4^ab^**	**0.94±0.1^hi^**	**1.96±0.04^a^**	**872±12^de^**	**1388±8.0^ab^**

Each value is a mean of three separate biological replicates, Mean ± Standard Deviation. Different parameters were analyzed using ANOVA to detect significant difference between means. Means were compared using Duncan’s Multiple Range Test (DMRT) at *P*<0.05. Different combinations of alphabets abcdefghi denote the significantly different groups.

### Reduced accumulation of ROS contents under cold stress

**Figure 5:**
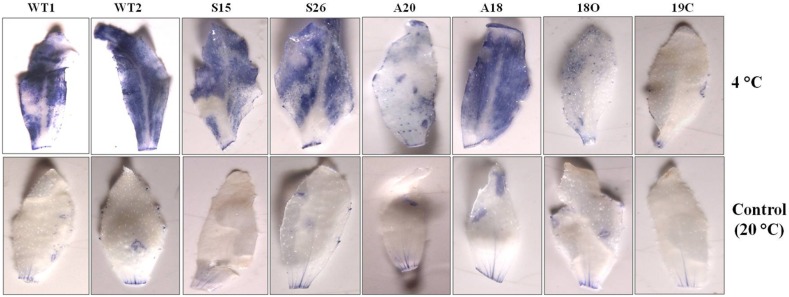
Histochemical staining assay of ROS accumulation with nitro blue tetrazolium (NBT) in the wild type and transgenics under cold stress. Leaves from potted plants that have been subjected to cold stress for 96 h were used for the accumulation of ROS.

Accumulation of ROS may cause damage to many bio molecules of the cells. As the transgenic lines were more tolerant to cold stress than WT, an attempt was made to compare accumulation of ROS in their leaf samples at the end of the cold stress by staining with NBT. Histo-chemical staining of leaves from WT and transgenic plant with NBT revealed that it could also be stained ROS without cold stress also (Fig. 5). The figure showed that the transgenic lines had slightly lower ROS accumulation relative to WT without stress. Cold resulted in significantly higher levels of ROS accumulation in WT leaves whereas the transgenic lines S26, S15, A20, A18, 18O and 19C low levels accumulation as evidenced by the lower intensity of the blue colour (Fig. 5). As expected, the double transgenic line 18O and 19C showed notable less blue colour than *PaSOD* (S26, S15) and *RaAPX* (A20, A18) transgenic plants. This implies that less ROS was accumulated in these transgenic lines.

### Accumulation of *PaSOD* and *RaAPX* in transgenic *Arabidopsis* improved the cold stress tolerance

**Figure 6:**
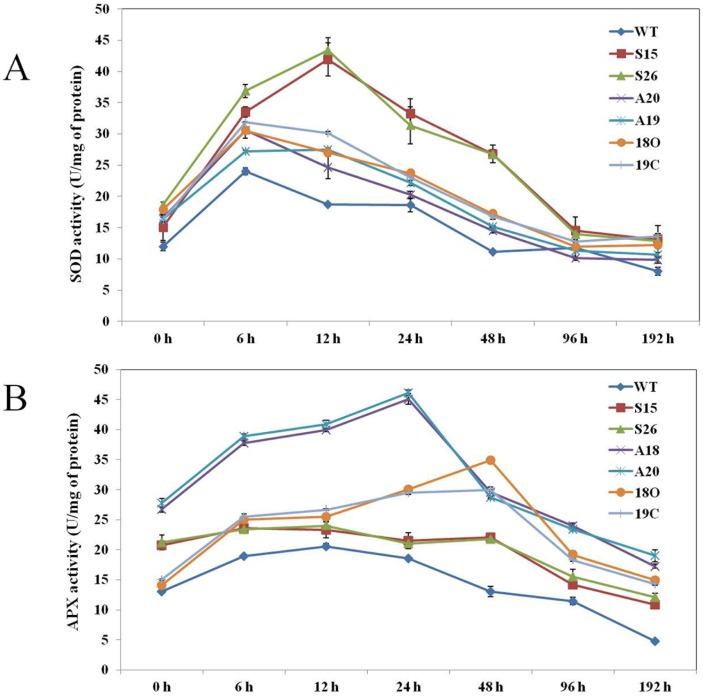
Estimation of total enzyme activities during cold stress both in WT and transgenics, A) superoxide dismutase and B) ascorbate peroxidase activity. Error bars represents ± SE of mean of three biological replicates.

As the main objective of the present study was to improve the scavenging of ROS by the over expression of supplemental antioxidant enzymes to the native ones, we checked the expression of the transgenes in the transgenics during cold stress. Superoxide dismutase and ascorbate peroxidase enzyme activities were estimated from the leaf samples of WT and transgenic plants at different time points during cold stress i.e. from 0–192 h under cold stress. As expected total SOD and APX activities were significantly higher in transgenic plants when compared to WT plants even under normal growth conditions, whereas 1.6, 1.2 and 1.3 folds increase in SOD levels was observed in SOD (S26, S15), APX (A20, A18) and double transgenic SOD and APX (18O, 19C) plants, respectively throughout 192 h of cold stress (Fig. 6). In the case of ascorbate peroxidase, 1.7-fold increase was recorded in transgenic plants A20 and A18 plants over WT plants under normal growth conditions and an increase of 2 fold was recorded throughout the cold stress. In double transgenic lines (18O and 19C) 1.3 folds higher activity was recorded. Abrupt elevation of these enzyme activities were recorded during the initial 12 h of cold in WT and all the transgenic lines and consequently a gradual decrease was recorded between 12 and 96 h, after which the minimal levels were maintained to sustain the growth and development of plants. Improved growth in terms of increase in biomass during cold stress in transgenic plants might be because of presence of the additional copy of antioxidant gene(s), implies increase in the activity of corresponding enzymes. Superoxide dismutase and ascorbate peroxidase enzyme activities were also estimated from the leaf samples of WT and transgenic plants at different time points without cold stress for comparisons. Slightly increased levels of SOD and APX activities were recorded in all the transgenic plants when compared to WT (Fig. S4).

### Over expression of antioxidant enzymes protects cellular membranes from freezing injury

**Figure 7:**
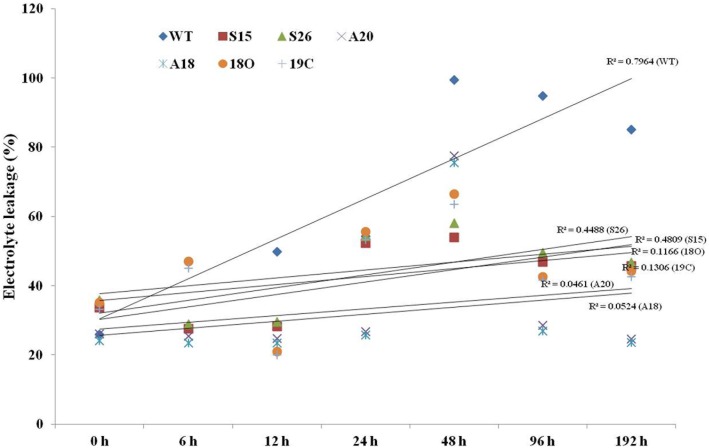
Change in relative electrolyte conductivity (electrolyte leakage) during cold stress in WT and transgenic plants. Error bars represents ± SE of mean of three biological replicates.

Stress accumulated ROS causes lipid peroxidation wherein the lipids in the cell membranes are damaged. Cold-induced cell membrane injuries in leaf tissues of transgenic lines were quantified by estimating the electrolyte leakage during *PaSOD* and *RaAPX* up regulation induced cold stress tolerance (Fig. 7). Chilling induced ion leakage was more in WT plants, when compared to transgenic plants. Whereas no difference was observed under normal conditions i.e. 20°C (Fig. S5). The relative electrolyte conductivity (REC) of transgenic plants was significantly lower than that of wild-type plants during cold stress. The results demonstrated that membrane damage was more in WT plants than that in the transgenic plants against cold stress. The ion leakage increased up to 48 h exposure to cold in *PaSOD*, *RaAPX* and double transgenic lines, but after that there was a decline. After prolonged exposure to cold the ion leakage is more prominent in WT (Fig. 7), whereas transgenics were less affected by the cold. It seems that the over expressing antioxidant enzymes might be protecting the membrane damage.

### Cold stress induces increase in aminoacid, soluble sugars and chlorophyll contents in transgenics

**Figure 8:**
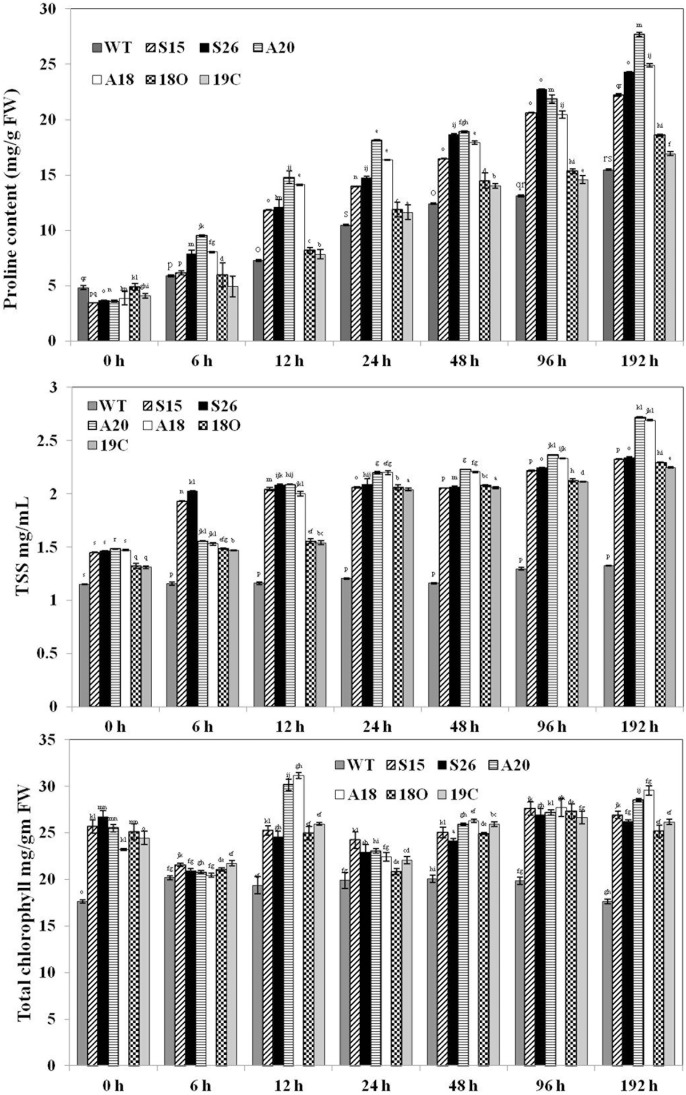
Estimation of different stress tolerance parameters in transgenics during cold stress (at 4°C). (A) Estimation of Proline content (B) Estimation of soluble sugars and (C) estimation of chlorophyll content. In all the cases, data is the mean of three biological replicates. Duncan’s multiple range tests was used to establish the statistical significance at *P*<0.05.

An increase in proline and simple sugars against stress is commonly known phenomena in *Arabidopsis* and other plants [28], [29], [30]. Under normal conditions, more or less similar amounts of proline content was recorded in all the plants, but higher levels of proline were found under stress in the transgenic lines when compared to corresponding WT plants (Fig. 8). Similar trend was observed in the case of soluble sugars under cold stress (Fig. 8). Overall, these findings suggested that the content of proline and soluble sugars, which are known osmo-protectants, accumulated more in transgenic plants under cold stress, thus providing better stress tolerance and better growth.

Chlorophyll content is susceptible to cold stress and usually continuously declines with cold stress [31]. In this study, chlorophyll content in the wild-type plants decreased at 4°C with the prolonged periods of cold stress. However, the transgenic plants have higher chlorophyll content than the WT plants under stress (Fig. 8). Thus cold tolerance of transgenic plants over expressing *PaSOD*, *RaAPX* and double transgenic plants showed increased chlorophyll accumulation.

### Cold stress tolerance by simultaneous over-expression of *PaSOD* and *RaAPX* is because of the accumulation of lignin in the vascular bundles

**Figure 9:**
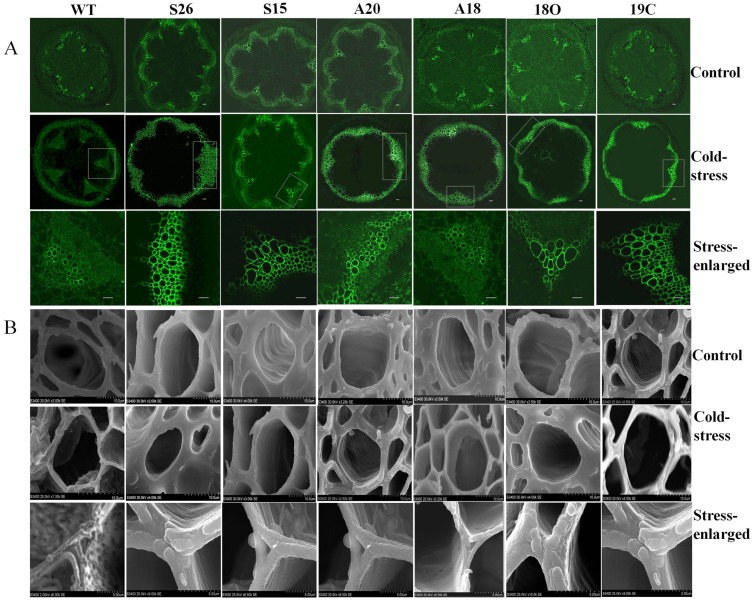
Pattern of lignin accumulation in transgenic *Arabidopsis* plants during cold stress. A) Transverse section of inflorescence stem of WT and transgenic lines (S26, S15, A20, A18, 18O and 19C) showing lignin accumulation pattern in WT and transgenic plants under cold stress. Close up view of the single vascular bundle of stress tissue is shown in the lower most panels (close view). Samples were collected after 192 h of the cold treatment. Lignin auto fluorescence was collected by excitation/emission wave lengths 488/505 nm by Confocal Laser Scanning Microscope. B) SEM analysis of vascular bundle walls showing thickness of lignin during cold stress.

Anatomical investigation of vascular changes during cold stress by using Confocal and scanning electron microscopy revealed that there was a dramatic alteration in lignin accumulation in transgenic plants as compared to the WT (Fig. 9A). Inter-fascicular lignification was evident under cold stress in single and double transgenics, whereas inflorescence stem of WT showed lignin deposition in the vascular bundles only (Fig. 9). Lignification of the inter-fascicular arcs was conspicuous along the entire stem and showed vascular bundles connected by lignified arcs in all transgenic lines, but more conspicuous in S26, S15 and 18O, 19C under stress condition only (Fig. 9). In addition, xylem elements of vascular bundles of the transgenic lines were larger than that of the WT plants.

The thickness of the walls of vascular bundles of the transgenic and WT were more or less the same (3µ) under normal conditions, whereas under cold stress the transgenic showed thicker walls. The thickness was more in double transgenics (18O and 19C) followed by *PaSOD* transgenics (S26 and S15) and *RaAPX* transgenics (A20 and A18) i.e. 12, 8 and 4 µ, respectively (Table 2).

**Table 2:** Details of the increase in lignifications of vascular bundle cell wall’s width during cold stress in transgenic *Arabidopsis* over expressing *PaSOD*, *RaAPX* and both.

Plant type	Width of the vascular bundle wall (µ) ±SE	
	Control (20°C)	Cold stress (4°C)
**WT**	**3±0.15**	**2±0.15**
***PaSOD*** ** (S26)**	**3±0.12**	**8±0.70**
***PaSOD*** ** (S15)**	**3±0.21**	**8±0.29**
***RaAPX*** ** (A20)**	**3±0.12**	**4±0.27**
***RaAPX*** ** (A18)**	**3±0.22**	**4±0.15**
***PaSOD*** ** + ** ***RaAPX*** ** (18O)**	**3±0.17**	**12±0.17**
***PaSOD*** ** + ** ***RaAPX*** ** (19C)**	**3±0.25**	**14±0.52**

Each value is a mean of three separate biological replicates.

### *PaSOD* and *RaAPX* induced cold tolerance modulated lignin biosynthesis pathway in transgenic *Arabidopsis*

**Figure 10:**
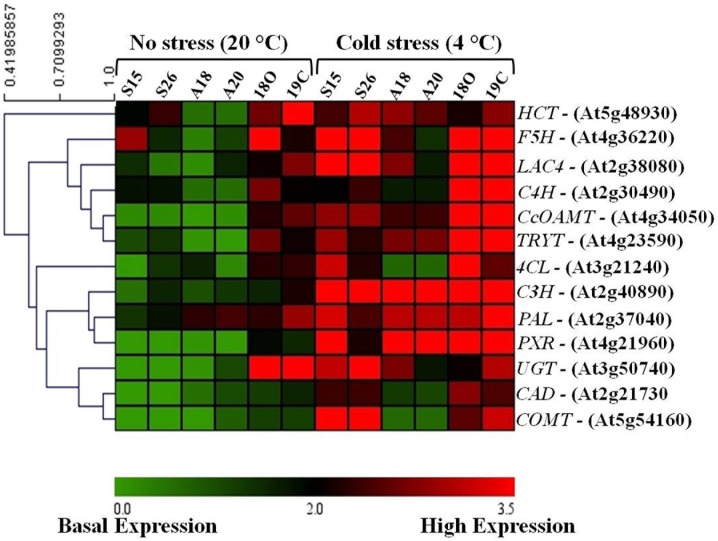
Heat Map of lignin biosynthesis pathway genes in transgenic *Arabidopsis* plants under cold stress (4°C). Phenylalanine ammonia lyase (*PAL1*), cinnamate 4-hydroxylase (*C4 H*), 4-coumarate:CoA ligase (*4CL-2*), ferulate 5-hydroxyase (*F5 H*), hydroxycinnamoyl-CoA transferase (*HCT*), *p*-coumarate 3-hydroxylase (*C3 H*), caffeoyl-CoA *O*-methyltransferase (*CCoAOMT1*), ferulate 5-hydroxylase (*F5 H*), caffeic acid *O*-methyltransferase (*COMT1*), cinnamyl alcohol dehydrogenase (*CAD2*), Laccase (*LAC4*), UDP-glucosyltransferase (*UGT 721 E1*) and Tyrosine ammonia lyase (*TRYT*) analysed in SOD, APX and double transgenic *Arabidopsis* plants.

Higher vascular lignin thickenings were observed in transgenics, it was assumed that increased H_2_O_2_ levels might be responsible for these accumulations. Lignin is a polymer where different subunits of lignin (4-hydroxy cinnamyl aldehyde, coniferyl alcohol and sinapoyl alcohol) are produced via phenylpropanoid pathway and polymerized by peroxidase (*AtPRX9GE*) in to lignin. To understand the possible alterations in lignin biosynthesis pathway with the simultaneous over expression of *PaSOD and RaAPX* in transgenics, the expression profiles of different genes of this pathway namely *COMT*, *CAD*, *4CL*, *TRYT*, *PXR*, *C4 H*, *CcOMAT*, *C3 H*, *HCT*, *LAC*, *UGT*, *F5 H* and phenylalanine ammonia-lyase 1 (*PAL1*) were assessed by real time PCR. Generated expression profile data confirmed that in transgenic lines an up-regulation of the expression of all these genes tested was recorded under cold stress as compared to the WT. The heat map shows an overview of expression profiles of the phenylpropanoid pathway genes, and their interactions are shown in Fig. 10. Almost all the genes tested showed a basal level expression in *PaSOD* (S15 and S26) and *RaAPX* (A18 and A20) transgenics, whereas, comparatively higher expression was recorded except *CAD, COMT, C3 H, PXR1* in double transgenics (18O and 19C) under normal conditions (20°C). An up regulation was observed in the expression of the core phenylpropanoid pathway genes, including *PAL*, cinnamate 4-hydroxylase (C4 H), and 4-coumarate CoA ligase (4CL) under cold stress. After that, the lignin pathway divides into three specific branches for the formation of H, G and S lignin monomers. Most of the identified genes involved in these three pathways were up-regulated during cold stress. *F5 H*, *LAC4*, *C3 H*, *PAL1*, *PXR1*, *CoMT1* were the commonly up regulated both in *PaSOD* and double transgenic plants. In *RaAPX* over-expressed lines i.e. A18 and A20 a basal level expression was recorded in *4CL2*, *CAD2*, *COMT1* even in cold stress. Overall, the results indicated that the activation of the phenylpropanoid pathway led to the synthesis of lignin in response to cold stress by the simultaneous over expression of *PaSOD* and *RaAPX*.

## Discussion

As compared to other living organisms, plants need to protect themselves from different environmental, abiotic and biotic stresses. These stresses cause oxidative damage, ion toxicity and disruption of cellular homeostasis through the production of reactive oxygen species (ROS). ROS induces various complex biochemical, molecular, cellular, and physiological changes in plants including damage of DNA, proteins and membrane lipids [14], [32]. The responses to abiotic stresses can generally be separated into two categories: those that regulate signal transduction and gene expression in response to the stress, and those that protect against environmental stress by redirection of metabolism. Resistance to cold and salt stress requires a number of changes in gene expression [33], [34], [35]. Plants have developed a wide range of enzymatic and non-enzymatic mechanisms to scavenge ROS. Among the enzymatic methods, superoxide dismutase (SOD) is one of the crucial enzymes in the plant’s defense mechanism that converts superoxide anion radicals (O_2_^−.^) to hydrogen peroxide (H_2_O_2_), there by imparting protection against the harmful effects of highly reactive O_2_^−.^ [14], [36]. This in turn with the help of other antioxidant enzymes such as APX and catalase converts them in to safer molecules like H_2_O and O_2_. The alpine climate is very harsh with low temperatures, the length of the daily photoperiod, and the quality of the incident light influences the plant growth in these areas. Because of having morphological, physiological and genetical adaptations to these stresses, the alpine vegetation can grow even in such harsh condition. So it is expected that these plants are known to have better enzymatic system whose activities are enhanced or are better adapted than their counterparts from plains. High altitude plants with efficient and active protective systems such as autoclavable superoxide dismutase from Potentilla [6], cell wall hydrolases with broad temperature range activity from Podophyllum [37], make these plants sustain under very low temperature conditions. Dissection of these adaptations in these plants has provided insights towards unraveling of the possible mechanisms of stress tolerance [14], [11]. Cold stress was shown to enhance the levels of different ROS-scavenging enzymes [38], [39] and accumulation of H_2_O_2_ in *Arabidopsis* callus cells was reported [40]. Scebba et al. [5] reported the induced expression of antioxidant enzymes SOD and CAT in wheat under cold stress. Over expression of *PaSOD* has improved salt and copper stress in *Arabidopsis thaliana* [11], [12].

Effects of low temperature stress includes reduced growth [41], [42], wilting [43], chlorosis [44], impaired photosynthesis, increased electrolyte leakage across membranes [45] and impaired reproductive development which ultimately reduces the yield [46], [47], [48], [49]. Reports were also available on successful manipulation of abiotic stresses with the simultaneous over expression of different antioxidant enzymes in Fescue plants, sweet potato and tobacco [50], [51], [52].

Present investigation dealt with the improvement of cold stress tolerance and studied the possible mechanism in *Arabidopsis* by the simultaneous over expression of antioxidant genes/enzymes *PaSOD* and *RaAPX* from the cold niche area plants. The present results showed that the WT plants were ‘chilling sensitive’ with stunted growth, reduced metabolism and with low biomass accumulation during cold stress. But the transgenic plants because of the manipulation of ROS detoxification mechanism by the over expression of antioxidant enzymes *PaSOD* and *RaAPX* performed better showing normal growth, more biomass, well developed root system, and higher yields in terms of more number of seeds per silique and thus have become ‘chilling tolerant’. In the present results are in agreement with the work of Yuanyuan et al. [53], where he shows that increased soluble sugars play an important role during cold acclimation process, in transgenic *Arabidopsis* plants and also accumulation of soluble sugars and proline accumulation was recorded in response to cold stress. Soluble sugars exert their positive effects to protect plant cells from damage caused by cold stress through several ways, including serving as osmo-protectants, nutrient as well as interacting with the lipid bi-layer. These changes might also be responsible for maintaining the membrane stability and thus leading to reduced electrolyte leakage in the transgenic plants. Superoxide dismutase induced membrane damage tolerance was also reported by Kwon et al. [54] in transgenic tobacco plants. The photosynthetic performance of transgenic *Arabidopsis* plants was commendable with higher chlorophyll content throughout the cold stress whereas the WT plants were not able to sustain the lesser chlorophyll. Perhaps, this might be the reason in slower/stunted growth of WT during stress.

Lignins are the secondary metabolites, which maintain the structural integrity and provide mechanical strength to the stem and also impart hydrophobicity to vasculature allowing water and nutrient transportation [55]. The ROS catalysed by SOD and gets reduced into H_2_O_2_ which has a versatile role in the plant metabolic system. These molecules at low concentration acts as mediator of signalling pathways leading to stress acclimation at higher concentration it orchestrates the cellular damage and death [40]. Hydrogen peroxide acts as secondary messenger in stress signalling pathways due to its long half life and high permeability across membranes [56], [57], [58]. In addition to this the elevated levels of H_2_O_2_ have been shown to induce polymerization of lignin monomers (H-, S- and G- lignin) in to lignin [59].

Lignin induction has been correlated with cold, drought, or light stresses as well as mechanical injuries in a large number of different plant species, such as poplar, rice, pine, *Arabidopsis*, and soybean [60]. In our earlier study also, over expression of *PaSOD* induced lignin accumulation in the vascular bundles under NaCl stress which has been demonstrated to be the molecular basis for improved stress tolerance [14]. In the present study, anatomical investigation of vascular structures using Confocal and electron microscopy clearly showed that accumulation of lignin in the walls of the vascular strands of transgenic plants with cold stress. Transgenic plant expressing *PaSOD* alone and double transgenics were more lignified than *RaAPX* and WT plants. This high deposition of lignin in *PaSOD* transgenics, is probably due to the over-accumulation of H_2_O_2_ produced by scavenging of over produced superoxide ion (O2^−.^) under cold stress. Over-accumulation of H_2_O_2_ is toxic [40] and subsequent increased lignification may lead to choking of vascular system, thus cause damage and cell death in WT. In double transgenics, detoxification mechanism of increased levels of H_2_O_2_ can be achieved by the over expression of extra copy of downstream enzyme i.e. APX. This reduces the over-accumulated H_2_O_2_ levels and also probably distributes towards cell-signaling and lignifications and hence better adaptation to withstand the stress conditions. In the present study, the real time transcript analysis using qPCR also confirmed the up regulation of lignin biosynthetic pathway. A clear up regulation the core enzymes of the phenylpropanoid pathway namely PAL, C4 H and 4CL, a clear up regulation was observed in transgenics under cold stress. After that the pathway divides in to three directions, each is responsible for the production of individual components of monomers of lignin polymer. It is possible that the up regulated genes might have a significant role in antioxidant induced lignification during cold stress.

In conclusion, the simultaneous over expression of *PaSOD* and *RaAPX* from high altitude plants improved cold stress tolerance in *Arabidopsis*. This stress tolerance might be because of over expression of highly evolved and cold adapted genes, namely *PaSOD* and *RaAPX* as evidenced by the enzymes assays, which also detoxifies ROS as evidenced by the *in situ* ROS staining of leaves. Increased accumulation of TSS, proline in transgenic plants during cold stress, probably might be involved in protecting the membrane integrity as a result low electrolyte leakage was observed. Transgenic *Arabidopsis* plants had long roots with higher biomass without compromising yield, which is consistent with improved stress tolerance. No significant increase in all these parameters under normal growth condition (20°C) was also additional evidence to state that the cold stress tolerance is because of the over-expression of *PaSOD* and *RaAPX* only. This work clearly demonstrates that the modulation of endogenous ROS scavenging capacity against abiotic stresses can be successfully engineered by the simultaneous over expression of *PaSOD* and *RaAPX* in *Arabidopsis*. In addition, the results outlined the importance of the cytosolic antioxidant machinery in the cross-protection from multiple stresses in agriculturally important plants.

## Supporting Information

Figure S1 Generalised scheme of lignin biosynthesis pathway. Each arrow shows a reaction in the pathway, and next to each arrow is the name of the enzyme that catalyzes the associated reaction, Abbreviations are listed as per pathway. CM, CHORISMATE MUTASE; PAT, PREPHENATE AMINOTRANSFERASE; AGT, AROGENATE DEHYDRATASE; PAL, PHENYLALANINE AMMONIA LYASE; C4H, CINNAMATE 4-HYDROXYLASE; 4CL, COUMARATE CoA LIGASE; CCR, CINNAMOYL-CoA REDUCTASE; CAD, CINNAMYL ALCOHOL DEHYDROGENASE; HCT, HYDROXYCINNAMOYL-CoA TRANSFERASE; C3H, *P*-COUMARATE 3-HYDROXYLASE; F5H, FERULATE 5-HYDROXYLASE; COMT, CAFFEIC ACID *O*-METHYLTRANSFERASE; UGT, UDP-GLUCOSYLTRANSFERASE; LAC, LACCASES; PXR, PEROXIDASES; CCoAOMT, CAFFEOYL-CoA 3-O-METHYLTRANSFERASE; H lignin, p-hydroxy phenyl lignin; G lignin, guaiacyl lignin monomers; S lignin, syringyl lignin monomers.
(TIF)

Figure S2 Growth of transgenic *Arabidopsis* plants under normal conditions (20°C) without cold stress. Five weeks old seedlings were transferred and allowed to grow at 20°C for 192 h and different stress tolerance parameters were estimated from the samples of these plants.
(TIF)

Figure S3 Root growth of WT and transgenic plants under normal conditions. A) Photograph showing root growth of transgenic plants without cold stress (192 h). B). Graphical representation of root length of transgenic plants without cold stress (192 h). Values are the representation of Mean±SE of three biological replicates. Transgenic plants developed better root system under normal growth conditions.
(TIF)

Figure S4 Estimation of total enzyme activities under normal growth conditions without cold stress both in WT and transgenics. A) superoxide dismutase and B) ascorbate peroxidase activity. Error bars represents ± SE of mean of three biological replicates.
(TIF)

Figure S5 Change in relative electrolyte conductivity (electrolyte leakage) in WT and transgenic plants under normal growth conditions. Error bars represents ± SE of mean of three biological replicates.
(TIF)

Table S1 Primer Sequence and PCR conditions for all the genes (including gene id and accession numbers) used for Semi-quantitative and Real Time expression analysis.
(DOCX)
