# Supplementary figures and images for: Simultaneous Over-Expression of PaSOD and RaAPX in Transgenic Arabidopsis thaliana Confers Cold Stress Tolerance through Increase in Vascular Lignifications
Source: PLoS One. 2014 Oct 17;9(10):e110302. doi: 10.1371/journal.pone.0110302 (PMC4201527; doi:10.1371/journal.pone.0110302)

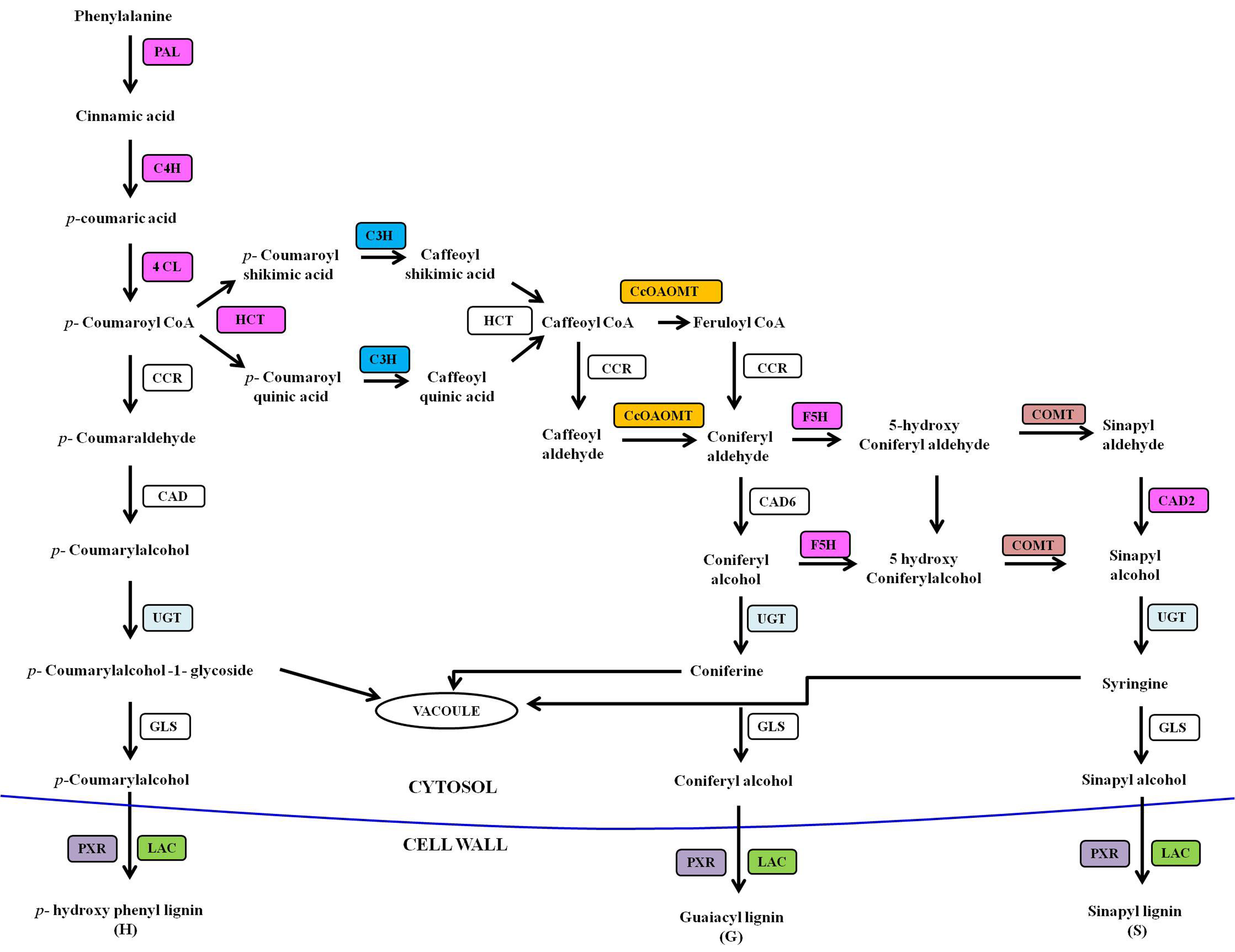

Supplement: Figure S1 — Generalised scheme of lignin biosynthesis pathway. Each arrow shows a reaction in the pathway, and next to each arrow is the name of the enzyme that catalyzes the associated reaction, Abbreviations are listed as per pathway. CM, CHORISMATE MUTASE; PAT, PREPHENATE AMINOTRANSFERASE; AGT, AROGENATE DEHYDRATASE; PAL, PHENYLALANINE AMMONIA LYASE; C4H, CINNAMATE 4-HYDROXYLASE; 4CL, COUMARATE CoA LIGASE; CCR, CINNAMOYL-CoA REDUCTASE; CAD, CINNAMYL ALCOHOL DEHYDROGENASE; HCT, HYDROXYCINNAMOYL-CoA TRANSFERASE; C3H, P-COUMARATE 3-HYDROXYLASE; F5H, FERULATE 5-HYDROXYLASE; COMT, CAFFEIC ACID O-METHYLTRANSFERASE; UGT, UDP-GLUCOSYLTRANSFERASE; LAC, LACCASES; PXR, PEROXIDASES; CCoAOMT, CAFFEOYL-CoA 3-O-METHYLTRANSFERASE; H lignin, p-hydroxy phenyl lignin; G lignin, guaiacyl lignin monomers; S lignin, syringyl lignin monomers. (TIF) [file pone.0110302.s001.tif]

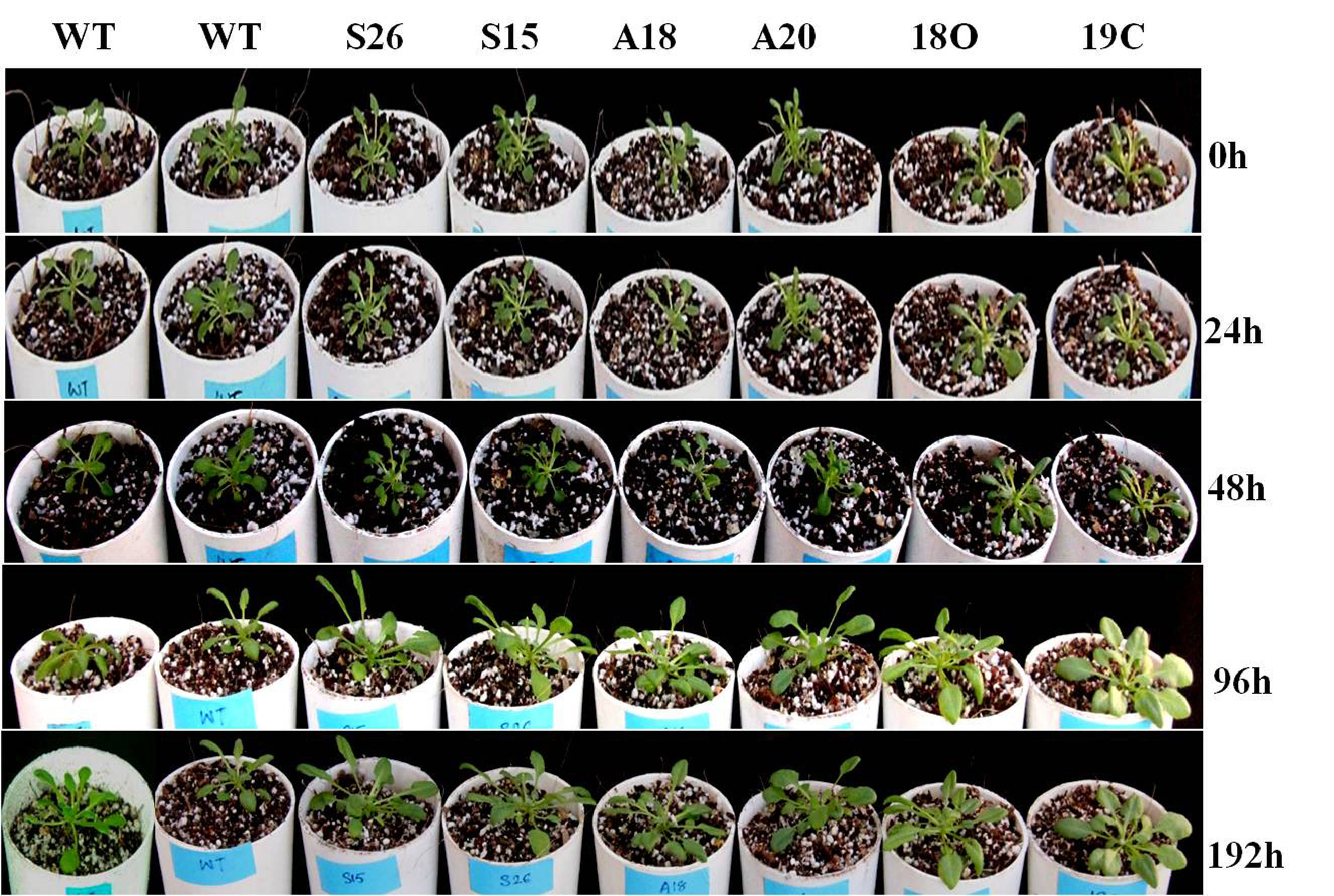

Supplement: Figure S2 — Growth of transgenic Arabidopsis plants under normal conditions (20°C) without cold stress. Five weeks old seedlings were transferred and allowed to grow at 20°C for 192 h and different stress tolerance parameters were estimated from the samples of these plants. (TIF) [file pone.0110302.s002.tif]

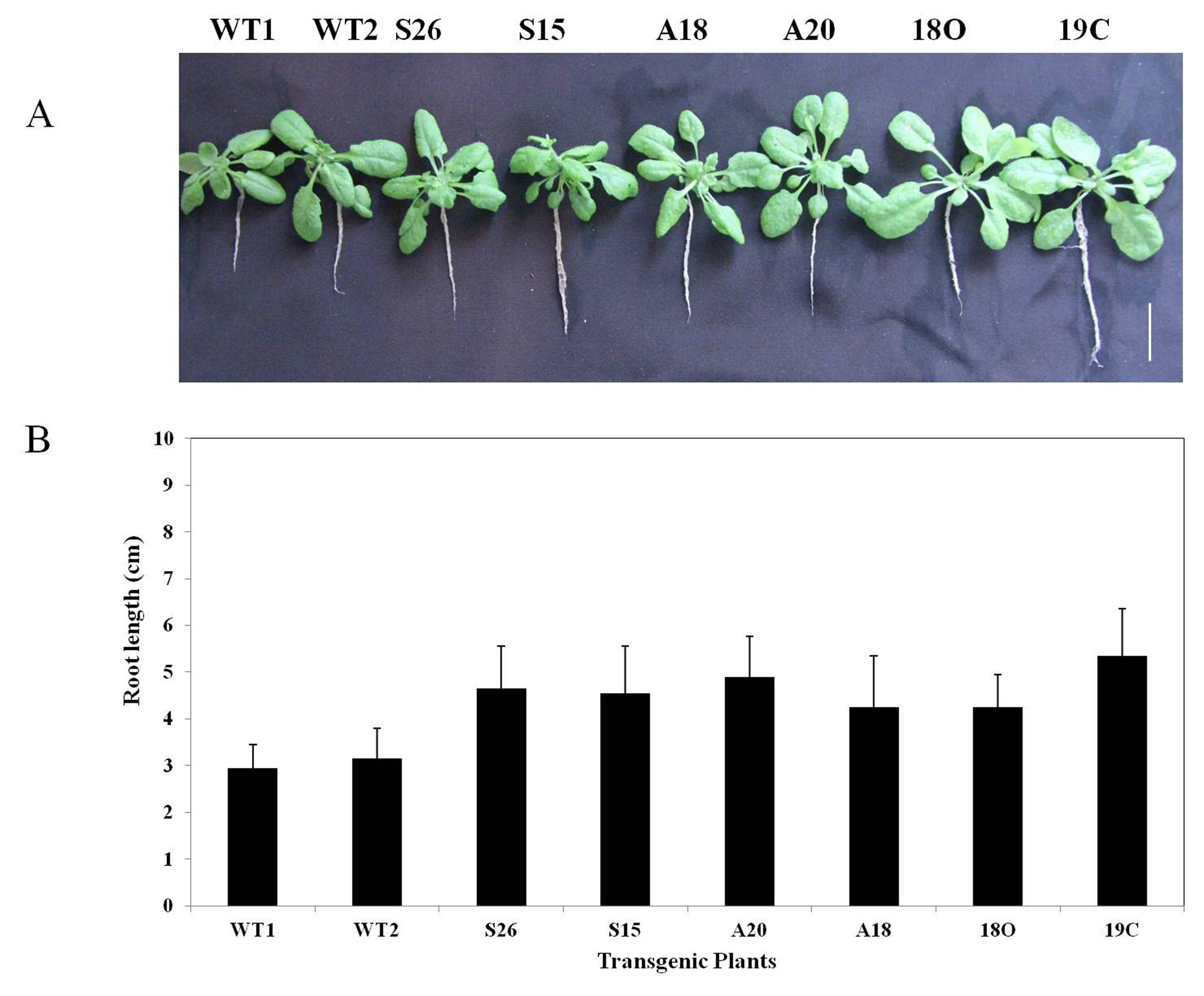

Supplement: Figure S3 — Root growth of WT and transgenic plants under normal conditions. A) Photograph showing root growth of transgenic plants without cold stress (192 h). B). Graphical representation of root length of transgenic plants without cold stress (192 h). Values are the representation of Mean±SE of three biological replicates. Transgenic plants developed better root system under normal growth conditions. (TIF) [file pone.0110302.s003.tif]

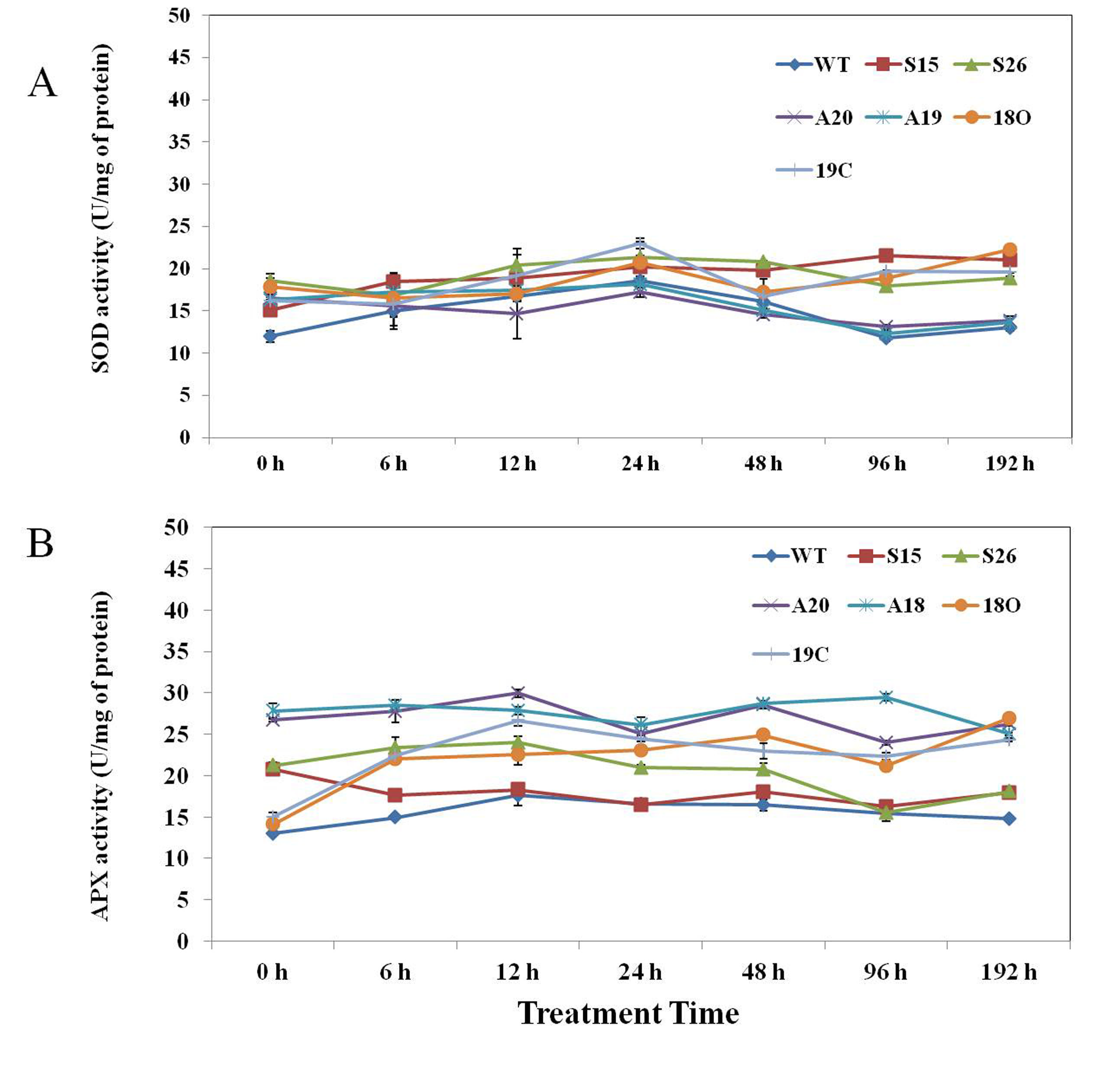

Supplement: Figure S4 — Estimation of total enzyme activities under normal growth conditions without cold stress both in WT and transgenics. A) superoxide dismutase and B) ascorbate peroxidase activity. Error bars represents ± SE of mean of three biological replicates. (TIF) [file pone.0110302.s004.tif]

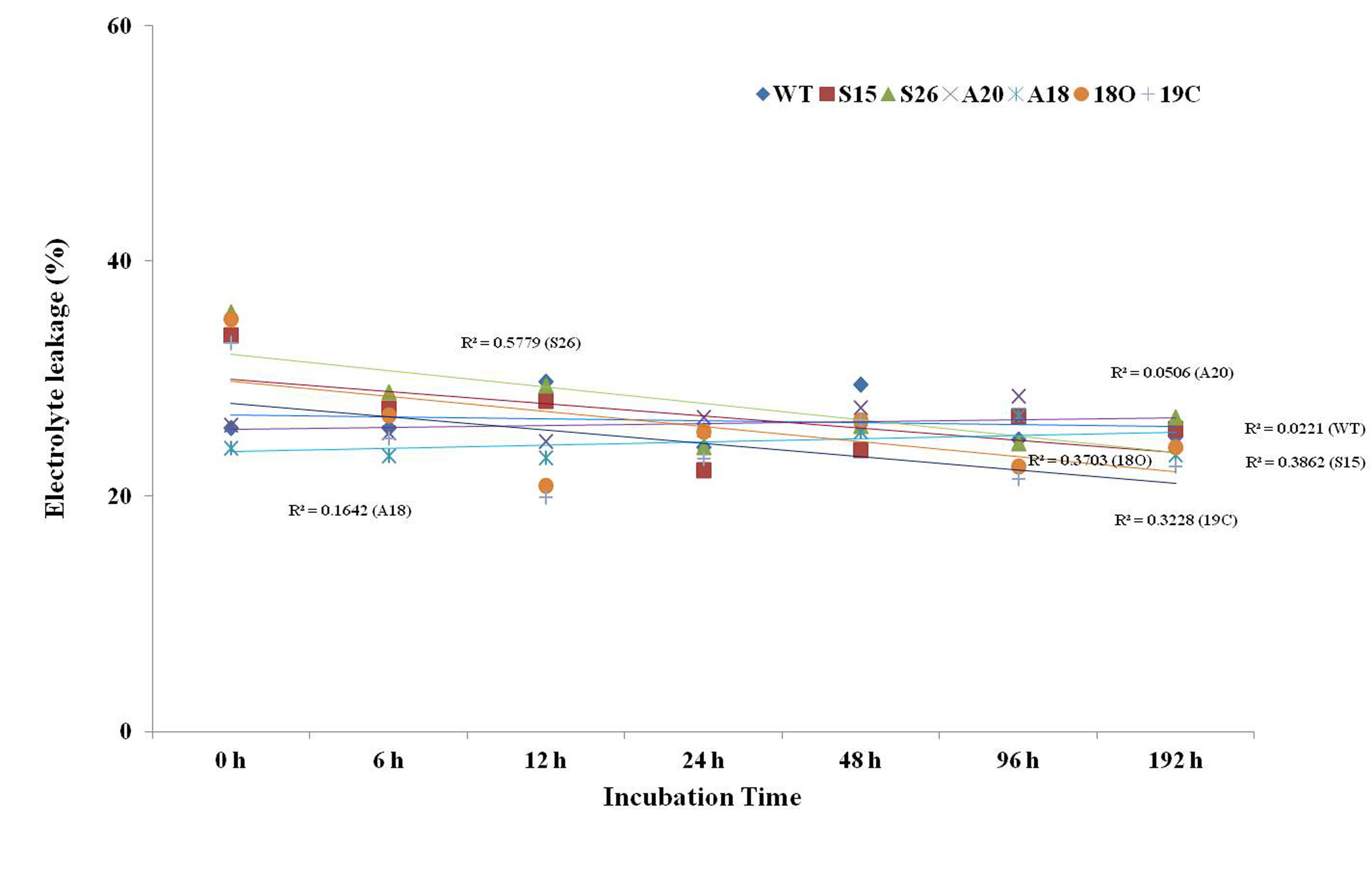

Supplement: Figure S5 — Change in relative electrolyte conductivity (electrolyte leakage) in WT and transgenic plants under normal growth conditions. Error bars represents ± SE of mean of three biological replicates. (TIF) [file pone.0110302.s005.tif]
