# Supplementary material for: Simultaneous Over-Expression of PaSOD and RaAPX in Transgenic Arabidopsis thaliana Confers Cold Stress Tolerance through Increase in Vascular Lignifications
Source: PLoS One. 2014 Oct 17;9(10):e110302. doi: 10.1371/journal.pone.0110302 (PMC4201527; doi:10.1371/journal.pone.0110302)
Supplement: Table S1 — Primer Sequence and PCR conditions for all the genes (including gene id and accession numbers) used for Semi-quantitative and Real Time expression analysis. (DOCX) [file pone.0110302.s006.docx]

**Table S1: Primer Sequence and PCR conditions for all the genes (including gene id and accession numbers) used for Semi-quantitative and Real Time expression analysis**

| Gene | Sequence 5'- 3' | | PCR conditions | Amplicon  size (bp) |
| --- | --- | --- | --- | --- |
| *PaSOD* | **F:**CCATGGATGGCAAAGGGCGTTGCTG **R:**TCTAGATCCTTGAAGGCCAATAATACC | | 94 ⁰C - 4 min; 94 ⁰C - 1 min,56 ⁰C - 30sec,72 ⁰C - 1 min, 27cycles; 72 ⁰C - 7 min | 456 |
| *RaAPX* | **F:**TGAGATCTGATGGCAGCGGCTCCAGTC **R:**GCACTAGTCTTCAGCTGCTTGCGAGC | | 94 ⁰C - 4 min; 94 ⁰C - 1 min,58 ⁰C - 30sec,72 ⁰C - 1 min, 27cycles; 72 ⁰C - 7 min | 855 |
| *AtSOD* | **F:** ATGGCGAAAGGAGTTGCAG  **R:** GCCCTGGAGACCAATGATG | | 94 ⁰C - 4 min; 94 ⁰C - 1 min,55 ⁰C - 30sec,72 ⁰C - 1 min, 27cycles; 72 ⁰C - 7 min | 456 |
| *AtAPX* | **F:** ATGGCTGCACCGATTGTT  **R:** CTTCATCCTCTTCCGGATCTC | | 94 ⁰C - 4 min; 94 ⁰C - 1 min,57 ⁰C - 30sec,72 ⁰C - 1 min, 27cycles; 72 ⁰C - 7 min | 861 |
| *GAP C* | **F:CTTGAAGGGTGGTGCCAAGAAGG**  **R:CCTGTTGTCGCCAACGAAGTCAG** | | 94 ⁰C - 4 min; 94 ⁰C - 1 min,55 ⁰C - 30sec,72 ⁰C - 1 min, 27cycles; 72 ⁰C - 7 min | 518 |
|  |  | |  |  |
| Primers used for real time expression analysis of Phenylpropanoid biosynthetic pathway | | | | |
| Gene  AGI Locus/  Accn No.(NCBI) | | **Sequence 5'- 3'** | **PCR conditions** | |
| *AtPAL1*  At2g37040/ NM_129260 | | **F:** TCATTGTAAGTTCTCAAATGAACAA  **R:** TCCGATGTTTGTTATGGATATTG | 95 ⁰C - 10 min (enzyme activation), 40 cycles each of 30 s at 95 °C, 30 s at 51 °C and 72 °C for 30 s | |
| *AtC4H* At2g30490/ NM_128601 | | **F:** CCCTTGTTTTGCTTTTGTGAG  **R:** TCCATCCAAAACAATGAGCAC | 95 ⁰C - 10 min (enzyme activation), 40 cycles each of 30 s at 95 °C, 30 s at 54 °C and 72 °C for 30 s | |
| *At4CL-2* At3g21240/ NM_113019 | | **F:** TGTTGTTGTTGCTGTTCTTGC  **R:** GTGCTGTTTTCTCCAGCACA | 95 ⁰C - 10 min (enzyme activation), 40 cycles each of 30 s at 95 °C, 30 s at 55 °C and 72 °C for 30 s | |
| *AtPXR1* At4g21960/ NM_118317 | | **F:** CGGAATGTGGTGTGTTATGG  **R:** CACATCATTCTTGGGTGCATA | 95 ⁰C - 10 min (enzyme activation), 40 cycles each of 30 s at 95 °C, 30 s at 54 °C and 72 °C for 30 s | |
| *AtTRYT* At4g23590/ NM_118490 | | **F:** CCCTCAAAGACGTCAATGGT  **R:** CCTCAAACCAAGAAACACTCA | 95 ⁰C - 10 min (enzyme activation), 40 cycles each of 30 s at 95 °C, 30 s at 54 °C and 72 °C for 30 s | |
| *AtCAD2* At2g21730/ NM_127743 | | **F:** TCCGGATCTAAGAATTGAGCA  **R:** AACCGAAAGCAGAAAACGAAG | 95 ⁰C - 10 min (enzyme activation), 40 cycles each of 30 s at 95 °C, 30 s at 54 °C and 72 °C for 30 s | |
| *AtCOMT 1*  At5g54160/ NM_124796 | | **F:** TTCACGAATTCTGCTACATGC  **R:** AAAGCAAACACCCTTTCACAAT | 95 ⁰C - 10 min (enzyme activation), 40 cycles each of 30 s at 95 °C, 30 s at 54 °C and 72 °C for 30 s | |
| *AtCCoAOMT1*  At4g34050/ NM_119566 | | **F:** GGATCTCCATGAAGCCAAGA  **R:** TGCGTTAGTGGTTACCATCC | 95 ⁰C - 10 min (enzyme activation), 40 cycles each of 30 s at 95 °C, 30 s at 55s °C and 72 °C for 30 s | |
| *AtACT2* At3g18780/ NP_188508 | | **F:** GATCTCCAAGGCCGAGTATG  **R:** CCCCAGCTTTTTAAGCCTTTG | 95 ⁰C - 10 min (enzyme activation), 40 cycles each of 30 s at 95 °C, 30 s at 55 °C and 72 °C for 30 s | |
| *AtUGT72E1* At3g50740/ NM_114934 | | **F:** CCGGACGAGTCGGTACTTTA  **R:** CCGTTGCTGACTCATCTCAA | 95 ⁰C - 10 min (enzyme activation), 40 cycles each of 30 s at 95 °C, 30 s at 55 °C and 72 °C for 30 s | |
| *AtLAC4* At2g38080/ NM_129364 | | **F:** TCATATGGTTTGGTTTCTATTTCTTG  **R:** AGCATAATCTAGTCACGTTCTTCA | 95 ⁰C - 10 min (enzyme activation), 40 cycles each of 30 s at 95 °C, 30 s at 55 °C and 72 °C for 30 s | |
| *AtC3H* At2g40890/ NM_180006 | | **F:** GCTAGAGACCCGGCTGTATG  **R:** ACGGAAGCAGCCTAAAATCA | 95 ⁰C - 10 min (enzyme activation), 40 cycles each of 30 s at 95 °C, 30 s at 55 °C and 72 °C for 30 s | |
| *AtHCT* At5g48930/ NM_124270 | | **F:** TTGTCATCCCCAGATTCCAT  **R:** GGCTTTGGAAAGAGCTTCCT | 95 ⁰C - 10 min (enzyme activation), 40 cycles each of 30 s at 95 °C, 30 s at 55s °C and 72 °C for 30 s | |
| *AtF5H* At4g36220/ NM_119790 | | **F:** GACCATCGAGGTTTTTGGAA  **R:** GCGCGTATAACCCTAGTTGC | 95 ⁰C - 10 min (enzyme activation), 40 cycles each of 30 s at 95 °C, 30 s at 55 °C and 72 °C for 30 s | |
